# Prevalence of delirium in German nursing homes: A cross-sectional study

**DOI:** 10.1186/s12877-026-07925-6

**Published:** 2026-07-10

**Authors:** Alexandre Houdelet-Oertel, Jonas Dörner, Roberto Walter, Zafer Arslan, René Müller-Widmer, Petra Thürmann, Horst Christian Vollmar, Ina Otte, Rebecca Palm, Bernhard Holle

**Affiliations:** 1 German Center for Neurodegenerative Diseases (DZNE), Witten, Stockumer St. 12, Witten, 58453 Germany; 2 School of Nursing Science, Faculty of Health, Witten/Herdecke University, Alfred- Herrhausen-St. 45, Witten, 58455 Germany; 3 Department of Medicine, Chair of Clinical Pharmacology, Faculty of Health, Witten/Herdecke University, Alfred-Herrhausen-St. 45, Witten, 58455 Germany; 4 Faculty of Medicine, Institute of General Practice and Family Medicine (AM RUB), Ruhr University Bochum, Universitätsstr. 150, Bochum, 44801 Germany; 5 Faculty of Medicine, Department of Health Services Research, Institute for Diversity Medicine, Ruhr University Bochum, Universitätsstr. 150, Bochum, 44801 Germany; 6 Carl von Ossietzky Universität Oldenburg School VI - School of Medicine and Health Sciences, Ammerländer Heerstraße 114-118, Oldenburg, 26129 Germany

**Keywords:** Cross-Sectional Studies, Delirium, Nursing homes, Prevalence, Risk Factors

## Abstract

**Background:**

Delirium is a common geriatric syndrome. However, there is a lack of primary data regarding the prevalence of delirium in German nursing homes. The aim of this study was to assess the prevalence of delirium and its motor subtypes, and to identify factors associated with the prevalence of delirium among residents of German nursing homes.

**Methods:**

This multicenter cross-sectional study was conducted in German nursing homes. Trained nurses collected data over a two-week period in April 2024. The participants were recruited via random sampling. The inclusion criteria were as follows: valid informed consent, age ≥ 65 years, and sufficient German language skills. The exclusion criteria were as follows: aphasia, coma, deafness, or end-of-life status. Delirium screening was conducted using the 4 ‘A’s Test (4AT), and delirium subtypes were classified using the Delirium Motor Subtype Scale (DMSS).

**Results:**

Data from 415 residents across 33 of the 34 participating nursing homes were included in the analysis. Probable delirium (4AT score ≥ 4) was identified in 17.6% of the residents (95% confidence interval [CI]: 13.4–22.7). The results of the DMSS revealed that 43.1% of individuals with delirium presented with a nonmotor subtype (95% CI: 29.8–55.9), 25.0% presented with a hypoactive subtype (95% CI: 15.6–33.3), 18.1% presented with a hyperactive subtype (95% CI: 12.0–24.4), and 13.9% presented with a mixed subtype (95% CI: 6.5–21.3). Multivariate logistic regression analysis revealed that more severe neuropsychiatric symptoms (adjusted odds ratio [AOR] = 1.17, 95% CI: 1.04–1.32), prevalent pain behavior (AOR = 3.67, 95% CI: 1.51–8.93), a medical diagnosis of dementia (AOR = 2.01, 95% CI: 1.12–3.59), and living in the nursing home for ≥ 25 months (AOR = 2.74, 95% CI: 1.08–6.94) were associated with the prevalence of delirium.

**Conclusion:**

The findings of this study confirm previous research indicating that delirium is a widespread health issue among residents of nursing homes. Further research is needed to develop training programs focused on the detection and prevention of delirium in nursing homes as well as to enhance knowledge regarding the delirium subtypes. The routine use of simple and rapid screening tools by nurses should also be integrated into standard care.

**Supplementary Information:**

The online version contains supplementary material available at 10.1186/s12877-026-07925-6.

## Introduction

Delirium is a common geriatric syndrome characterized by acute disturbances in attention, awareness, and cognition that develop rapidly, represent a sudden deviation from baseline functioning, and fluctuate in severity throughout the day [1]. The diverse clinical presentations of delirium, involving various hyperactive and hypoactive symptoms [2] that overlap with other conditions, such as dementia and depression [3, 4], make this syndrome highly heterogeneous and difficult to recognize by health care professionals [5–7].

Delirium is associated with serious negative outcomes, including prolonged hospital stays, increased risk of institutionalization, decreased cognitive ability, and increased mortality rates; furthermore, delirium can cause distress in affected individuals, relatives, and healthcare professionals [8–11]. Early recognition of delirium and its potential causes is important, as there is evidence suggesting that appropriate interventions may help reduce the severity and duration of delirium episodes [12], which in turn might be associated with improved outcomes [13]. Delirium represents an acute cerebral response to underlying physical etiologies, including systemic illness as well as pharmacological, iatrogenic, or surgical factors [14]. The exact pathophysiological mechanisms underlying the development of delirium have yet to be fully elucidated, but several neurobiological processes have been suspected to contribute to its pathogenesis [15].

Residents of nursing homes are typically older, often have multiple comorbidities, and are frequently affected by dementia and functional impairments [16, 17], all of which are recognized as predisposing factors for the development of delirium. A recent systematic review reported a wide range in delirium prevalence in nursing homes (1.0%–57.9%). The pooled estimate was 18.8%, although substantial heterogeneity was observed, likely due to differences in the prevalence of dementia and delirium detection methods [18]. When focusing on prospective prevalence studies from the past decade, reported prevalence rates range between 14.2% [19] and 36.8% [17]. 

To the best of our knowledge, no studies from Germany have estimated the prevalence of delirium or investigated associated factors using primary data collected in nursing homes. However, such data could raise greater awareness of this health issue among healthcare professionals and nursing home administrators, potentially prompting targeted measures for prevention, early detection, staff training, and structured delirium management. Therefore, the primary objective of the present study was to estimate the prevalence of delirium and its subtypes in German nursing homes. The secondary aim of the present study was to identify factors associated with the occurrence of delirium.

## Methods

The prevalence of delirium and its associated factors was estimated using primary data collected by trained nurses with standardized assessment tools as part of a multicenter cross-sectional observational study conducted in nursing homes in the federal state of North Rhine-Westphalia, Germany. This study is part of the Delirium in Nursing Homes (DeliA) consortium project [20] (https://delia.info). The study was reported in accordance with the Strengthening the Reporting of Observational Studies in Epidemiology (STROBE) guidelines for observational research [21]. As the detailed study methodology has been published previously [22], a brief summary is provided where appropriate.

### Settings and participants

All nursing homes in the federal state of North Rhine-Westphalia, Germany, as defined by the German Social Code XI (§ 71, paragraph 2), with at least 50 residents and not exclusively caring for specific groups, were considered potentially eligible. At least one registered nurse from each participating nursing home served as a rater. These nurses were required to have a minimum of three years of professional education, and they were responsible for recruiting residents and conducting data collection within the nursing home where they worked. Residents aged ≥ 65 years who received long-term care and had sufficient German language skills to understand and respond to the rater’s questions were eligible to participate. Participation in the study required residents, or their legal representatives (if self-determination was not possible), to provide informed consent. Residents with end-of-life status, residents in a coma, residents who were deaf (despite hearing aids) or residents who had aphasia were excluded.

### Sample size

To ensure sufficient power in the multivariate logistic regression analysis of observational studies, the sample size was calculated a priori using the following formula proposed by Bujang et al. [23]: *n* = 100 + 50$\:i$; where $\:i$ denotes the anticipated number of independent variables to be included in the final model. Assuming 11 independent variables, the minimum required sample size was estimated at 650 residents. To account for potential dropouts, the target sample was increased to 750 residents. To minimize structural effects within the nursing homes, the data collection was limited to 15 residents per home, thus requiring the participation of 50 nursing homes.

### Recruitment

First, the goal was to recruit 50 nursing homes where data collection was conducted, using opportunities identified via email, telephone, and trade journals. The second goal was to recruit 15 residents from each participating nursing home between February 2024 and the start of data collection in April 2024; a random sampling approach was used to minimize selection bias. For each nursing home, a recruitment list of 30 randomly ordered residents was created from the full resident population. If the initial list was insufficient to meet the recruitment target, it was expanded by 10 residents at a time. Using this list, the raters screened the residents sequentially for eligibility. Eligible residents were approached by the raters in the given order and informed about the study, both orally and in writing. For residents with legal representatives, the representatives were also informed. Informed consent was obtained from the participants or their legal representatives upon their agreement to participate.

### Training of the raters

To minimize measurement bias, all the raters received training provided by the study team, which included two nursing scientists and a general practitioner, all with expertise in delirium care, as well as a pharmacist. The training was initially conducted in one-day sessions at central locations, covering medical and pharmacological knowledge about delirium, as well as study management. The study management section of the training focused on handling recruitment and data collection, particularly administration of the 4 “A’s” Test (4AT) [24], supported by case studies on its assessment. For raters who were unable to attend these sessions, online training covering the study management components of the in-person training was provided. All raters received the same training manual containing detailed guidance on study procedures and data collection.

### Data collection

The data were collected using a standardized paper and pencil approach between April 15 and April 28, 2024, by the raters for all the residents who provided informed consent. The first step of data collection involved conducting delirium assessments using the 4AT, followed immediately by functional and health assessments for each resident. These assessments could be performed at any time during the data collection period. Once the assessments were completed, the raters collected data from the residents’ nursing records, which were restricted to entries made up to the day the assessments were conducted. Furthermore, to describe the characteristics of the participating rater, sociodemographic data were collected by the study team. Structural data about the participating nursing home were gathered from the nursing home management by the study team.

### Measurements

#### Delirium assessments

Delirium was assessed using the 4AT, a proxy-rated screening that is considered quick (< 2 min) and easy to administer [24]. The 4AT consists of 4 items (assessing the level of alertness, attention, orientation, and acute change or fluctuation course) and creates a sum score of 0–12. A score of ≥ 4 indicates possible delirium [24]. The 4AT has shown good diagnostic results in several clinical populations, with a pooled specificity of 88% and a pooled sensitivity of 88% [25]. The 4AT is recommended for application in long-term care settings [26] and has previously been employed in prevalence studies conducted in nursing homes [17, 26, 27].

To assess the motor subtypes of delirium, the Delirium Motor Subtype Scale (DMSS) [28] was applied if the 4AT screening indicated possible delirium (score of ≥ 4). The DMSS consists of 11 items rated as either present or absent, with items 1–4 representing hyperactive symptoms and items 5–11 representing hypoactive symptoms. The following subtypes are evaluated by the DMSS: the hyperactive subtype is identified if at least two items from 1 to 4 are positive; the hypoactive subtype is identified if items 5 or 6 are positive in addition to at least one item from items 7–11; the mixed subtype is identified if both criteria are met; and the nonmotor subtype is identified if none of the criteria are fulfilled. Depending on the subtype, the instrument exhibits a specificity ranging from 81.6% to 100% and a sensitivity ranging from 22.2% to 96.7%, indicating good diagnostic accuracy across subtypes [28].

#### Health and functional assessments

The Dementia Screening Scale (DSS) [29] was used to assess cognitive impairments. The DSS, developed for use by nurses, assesses two domains of cognitive functioning (memory and orientation) and consists of seven items. The total score ranges from 0 to 14, with higher scores indicating more severe impairment [29]. For this analysis, a cutoff score of ≥ 3 was applied to identify possible dementia (specificity: 90.9%; sensitivity: 81.4%) [29], and the total score served as a measure of cognitive impairment severity.

To assess functional impairments, the Physical Self-Maintenance Scale (PSMS), a valid and reliable measurement for older people with items related to activities of daily living (ADLs), was used [30]. The items for the ADLs (such as toilet or bathing) were rated on a scale ranging from not dependent to completely dependent [30], yielding a possible total score of 6–30 in this study, which was used as a continuous variable.

Nutritional status was assessed using the Mini Nutritional Assessment Short-Form (MNA-SF) [31], which consists of six items and can be completed using either body mass index (BMI) or calf circumference (CC). The MNA-SF categorizes individuals into three groups as follows: 12–14 points (normal nutritional status), 8–11 points (risk of malnutrition), and 0–7 points (malnutrition). The MNA-SF has been validated in various elderly populations and has been shown to exhibit good sensitivity and specificity [31].

To assess pain from the proxy-rating perspective, the German Version [32] of the Pain Assessment in Advanced Dementia (PAINAD) [33] was used for all residents. This scale consists of five items reflecting observed behaviors related to pain (such as negative vocalization). A total score between 0 and 10 points can be determined, with higher scores indicating more severe pain [33]. For this analysis, a score of ≥ 2 was used to indicate probable pain. This cutoff value has been validated in older individuals with dementia, with a specificity of 93% and a sensitivity of 77% [34]. Additionally, the total score was used as a measure of pain severity. The Numerical Rating Scale (NRS) for pain – a self-reported measure as the “gold standard” for pain assessment [35] – was administered to residents deemed capable of providing a valid self report of pain, as judged by the rater. In this study, the NRS score ranged from 0 to 10, with higher scores indicating greater pain. Among the residents able to self-report, the total score was used as an additional variable to assess pain severity.

Furthermore, neuropsychiatric symptoms were assessed using the Neuropsychiatric Inventory-Questionnaire (NPI-Q) [36], which consists of 12 items that reflect symptoms such as hallucinations and agitation/aggression. In the present study, the rating referred to the presence of neuropsychiatric symptoms with 0–12 points and their severity with 0–36 points (both total scores). Among individuals with probable dementia, the NPI-Q has been shown to have adequate reliability and a strong correlation with the well-validated original Neuropsychiatric Inventory (NPI) (total score, *r* = 0.91) [36]. Both total scores were used as continuous variables.

#### Additional resident characteristics

Additional sociodemographic, clinical, and care-related information was obtained from the residents’ documentation. For the analysis, the following variables were defined: age in years; sex (female or male); family status (widowed, divorced, single, married or in a partnership); and length of stay in the nursing home (≤ 24 months or ≥ 25 months). The Charlson Comorbidity Index (CCI) was calculated to assess the burden of multimorbidity on the basis of International Classification of Diseases, 10th Revision, German Modification 2024 (ICD-10-GM 2024) diagnoses and using the updated version reported by Quan et al. (0–24 total score) [37, 38]. Further binary variables (yes/no) were included: history of hospital stays in the past 3 months; vision impairment (despite the use of vision aids); hearing impairment (despite the use of hearing aids); falls in the past 3 months; use of freedom-depriving measures in the past 4 weeks (e.g., bed rails or belt restraints); presence of a feeding tube; presence of a urinary catheter; administration of neuroleptics (defined as the use of at least one Anatomical Therapeutic Chemical Classification System (ATC) N05A neuroleptic, irrespective of chronic or acute treatment); and diagnoses – defined on the basis of ICD-10-GM 2024 – of dementia (F00-F03, G30, G31.0, and G31.82), depression (F32-F33 and F34.1), Parkinson’s disease (G20-G22 and G23.2), and hypertension (I10-I13 and I15).

### Statistical analysis

All the statistical analyses were performed using R (v4.5.1; R Core Team 2025 and RStudio IDE (v2025.05.1; Posit Software, PBC). The threshold for statistical significance was set at α = 5%. Continuous variables are presented as the means with standard deviations (SDs) for normally distributed data and medians with interquartile ranges (IQRs) for nonnormally distributed data. Categorical variables are presented as absolute numbers (n) and relative frequencies (%). Group comparisons between residents with and without delirium (4AT score ≥ 4 versus < 4) were conducted using independent-samples t tests or Mann–Whitney U tests for continuous variables and Chi-square tests (χ²) or Fisher’s exact tests for categorical variables, depending on data distribution. DSS variables were included only in group comparisons because a documented diagnosis of dementia was considered a more appropriate indicator of pre-existing cognitive impairment and is less likely to be influenced by current cognitive status.

The regression analysis followed a two-stage variable selection strategy. Univariate logistic regression models were fitted for variables that showed significant group differences. Variables that were significant in the univariate analyses were entered simultaneously into a forced-entry multivariate logistic regression model adjusted for age and sex. To minimize the risk of bias due to structural differences across nursing homes, all regression analyses and delirium prevalence estimates used cluster-robust standard errors. This approach was chosen over multilevel logistic regression because cluster sizes were small and highly variable, ranging from 1 to 15 residents per nursing home, and our primary interest was to estimate overall resident-level associations rather than between-facility variance. Under these highly unbalanced cluster-size conditions, estimation of facility-level random effects may be unstable or of limited value [39]. For the multivariate model, common statistical assumptions (including multicollinearity, residual and outlier analyses) and goodness-of-fit criteria (pseudo R^2^, Brier score, and receiver operating characteristic curve [ROC curve]) were thoroughly assessed and confirmed to meet acceptable standards [40].

### Protocol deviations

Assessments were initially planned to be completed within five consecutive days to minimize varying conditions within nursing homes. However, due to high workloads, some nursing homes needed extra time. To prevent a substantial reduction in sample size and potential loss of statistical power, the period was retrospectively extended to two weeks. Sensitivity analysis confirmed that the prevalence of delirium and the effects were similar between the 5-day and 2-week samples (Supplementary material A and B).

## Results

**Fig. 1:**
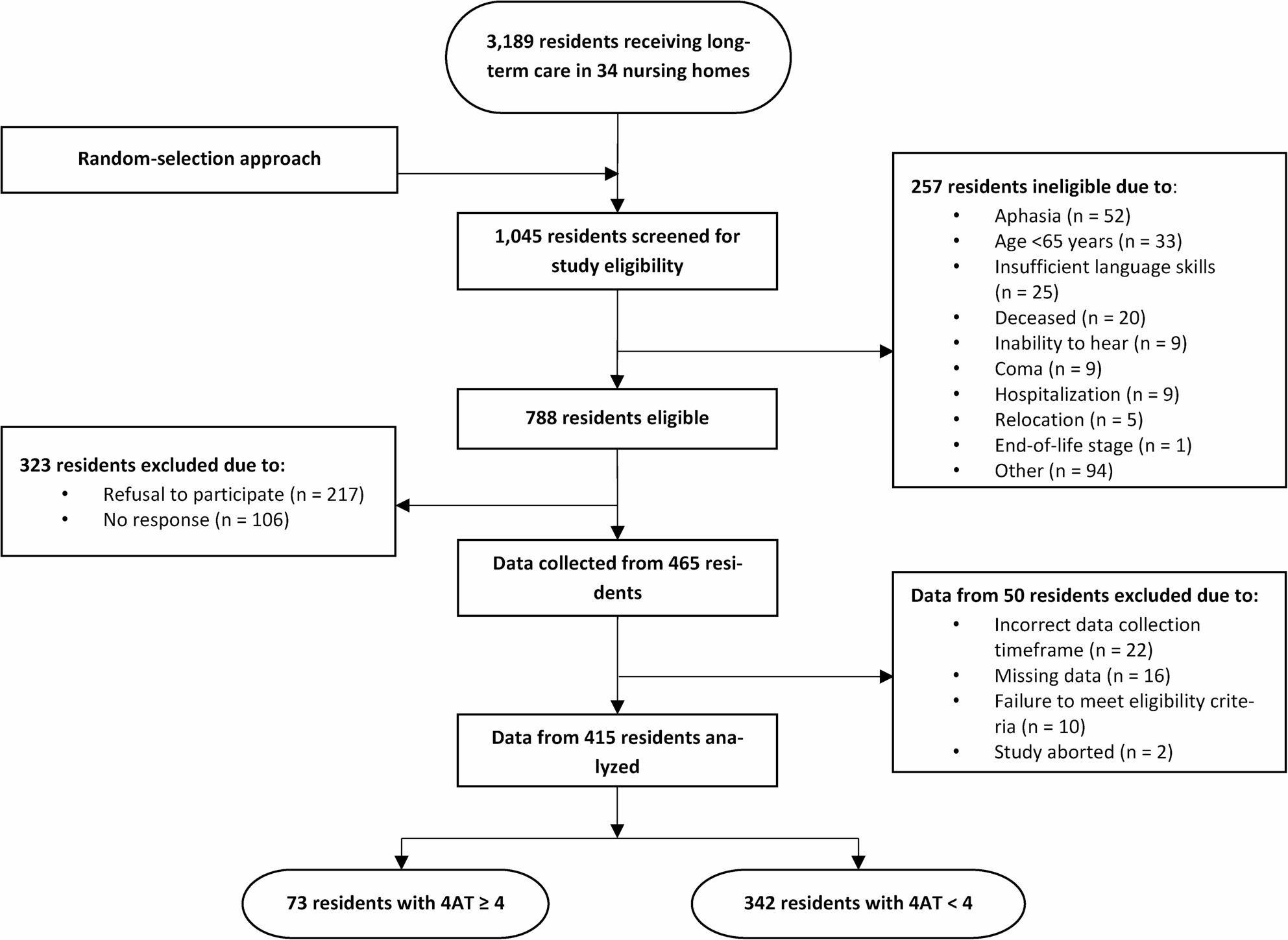
Flowchart of sample selection

Initially, 50 nursing homes were recruited. Nine nursing homes withdrew before data collection, and an additional seven nursing homes did not collect any data, resulting in 34 nursing homes that provided data. Among nursing homes that provided reasons for withdrawal, reported reasons included staff shortages and changes in staffing structures. At the start of recruitment in February 2024, these nursing homes cared for 3,189 long-term care residents, and 1,045 residents were randomly screened. Among the 788 eligible residents, 465 residents provided consent. However, one nursing home was excluded because resident data were not collected within the 2-week data collection period. Consequently, data from 415 residents across 33 nursing homes were ultimately included in the analysis (Fig. 1). The characteristics of the included nursing homes and their raters are presented in Table 1.

**Table 1:** Characteristics of the included nursing homes and participating raters

Variable		Measurement	*n*	Sample results
	**Characteristics of included nursing homes (** ***n*** ** = 33)**			
Nursing home funding		Nonprofit, n (%)	33	33 (100.0)
		For-profit, n (%)	0	0 (0.0)
Living and nursing units		Units, mean (SD)	32	4.1 (1.9)
Available long-term care places^a^		Places, median (IQR)	33	83.0 (78–112)
Long-term care residents		Residents, median (IQR)	33	81.0 (73–107)
Raters per nursing home		Rater, mean (SD)	33	1.4 (0.6)
	**Characteristics of participating raters (** ***n*** ** = 45)**			
Age		Years, mean (SD)	43	39.7 (10.9)
Female sex		Yes, n (%)	44	31 (70.5)
Employment in current nursing home		Years, median (IQR)	44	6.9 (2–10)
Total work experience in nursing^b^		Years, median (IQR)	43	12.0 (10–24)
Received in-person training		Yes, n (%)	45	41 (91.1)
Received online training		Yes, n (%)	45	4 (8.9)

Definitions of abbreviations: IQR Interquartile range, *SD* Standard deviation

^a^ Definition includes intermittent short-term care

^b^ The definition includes a training period, which usually lasts 3 years in Germany

The nursing home residents in the total sample had a mean age of 86.2 ± 8.0 years, with 74.2% being female residents and 40.3% (167/414) being residents with a documented dementia diagnosis in their records. Probable delirium (a 4AT score ≥ 4) was indicated in 73 nursing home residents, corresponding to a prevalence of 17.6% (95% confidence interval [CI]: 13.4–22.7). DMSS data were available for 72 residents because one DMSS assessment was incomplete. According to the DMSS, 13 residents had the hyperactive subtype (18.1%; 95% CI: 12.0–24.4), 18 had the hypoactive subtype (25.0%; 95% CI: 15.6–33.3), 10 had the mixed subtype (13.9%; 95% CI: 6.5–21.3), and 31 had the nonmotor delirium subtype (43.1%; 95% CI: 29.8–55.9).

### Characteristics of residents with and without delirium

A comparison of residents with and without delirium across all the included measures revealed certain significant differences (Table 2). Residents with prevalent delirium more often lived in the nursing home for 25 months or longer, showed greater limitations in daily activities (PSMS), exhibited more frequent and pronounced cognitive impairment (DSS), and were accordingly more frequently diagnosed with dementia. Residents with delirium were more likely to be at risk of, or already affected by, malnutrition (MNA-SF), exhibited more prevalent and severe pain behavior (PAINAD), had more often and more severe neuropsychiatric symptoms (NPI-Q), and received neuroleptics more frequently.

**Table 2:** Characteristics of residents with and without delirium

Variable		Measurement	*n*	Delirium	No delirium	*p*	ES
	**Sociodemographic characteristics**						
Age		Years, mean (SD)	415	86.4 (7.3)	86.1 (8.1)	.775^a^	0.03
Sex of resident		Female, n (%)	415	59 (80.8)	249 (72.8)	.203^b^	0.07
		Male, n (%)		14 (19.2)	93 (27.2)		
Family status		Widowed, n (%)	413	43 (59.7)	217 (63.6)	.513^b^	0.07
		Divorced, n (%)		8 (11.1)	23 (6.7)		
		Single, n (%)		12 (16.7)	48 (14.1)		
		Married, relationship, n (%)		9 (12.5)	53 (15.5)		
Length of stay in current nursing home		≤ 24 months, n (%)	400	27 (37.0)	196 (59.9)	**0.001** ^**b**^	**0.18**
		≥ 25 months, n (%)		46 (63.0)	131 (40.1)		
Hospital stay(s) < 3 months		Yes, n (%)	398	9 (12.9)	42 (12.8)	1.00^b^	< 0.01
	**Health and functional assessments**						
DSS score		Score 0–14, median (IQR)	409	11.0 (8–13)	3.0 (1–7)	**< 0.001** ^**d**^	**0.43**
DSS cutoff		Mild to severe dementia: ≥ 3, n (%)	409	70 (97.2)	209 (62.0)	**< 0.001** ^**c**^	**0.29**
PSMS score		Score 6–30, mean (SD)	410	20.6 (4.5)	15.3 (5.4)	**< 0.001** ^**a**^	**1.00**
MNA-SF cutoff		Normal: Score 12–14, n (%)	404	11 (15.1)	134 (40.5)	**< 0.001** ^**b**^	**0.25**
		Risk: Score 8–11, n (%)		42 (57.5)	164 (49.5)		
		Malnutrition: Score 0–7, n (%)		20 (27.4)	33 (10.0)		
PAINAD score		Score 0–10, median (IQR)	392	2.0 (0–4)	0.0 (0–1)	**< 0.001** ^**d**^	**0.33**
PAINAD cutoff		Probable pain: ≥ 2, n (%)	392	42 (59.2)	67 (20.9)	**< 0.001** ^**b**^	**0.33**
NRS (Pain) score		Score 0–10, median (IQR)	370	0.0 (0–2)	0.0 (0–2)	.779^d^	0.01
NPI-Q symptom score		Score 0–12, median (IQR)	406	3.0 (2–6)	1.0 (0–2)	**< 0.001** ^**d**^	**0.31**
NPI-Q severity score		Score 0–36, median (IQR)	399	4.5 (2–10)	1.0 (0–3)	**< 0.001** ^**d**^	**0.34**
	**Clinical and care-related characteristics**						
Vision impairment^e^		Yes, n (%)	413	20 (27.8)	101 (29.6)	.866^b^	0.02
Hearing impairment^e^		Yes, n (%)	410	24 (33.3)	84 (24.9)	.182^b^	0.07
Fall(s) < 3 months		Yes, n (%)	405	21 (30.0)	74 (22.1)	.206^b^	0.07
Physical restraint < 4 weeks^f^		Yes, n (%)	415	0 (0.0)	6 (1.8)	.596^c^	0.06
Feeding tube		Yes, n (%)	415	0 (0.0)	5 (1.5)	.592^c^	0.05
Urinary catheter		Yes, n (%)	415	3 (4.1)	28 (8.2)	.327^c^	0.06
CCI score		Score 0–24, median (IQR)	414	2 (2–3)	2 (1–3)	.310^d^	0.05
Neuroleptics		Yes, n (%)	413	45 (61.6)	127 (37.4)	**< 0.001** ^**b**^	**0.19**
Dementia		Yes, n (%)	414	43 (58.9)	124 (36.4)	**0.001** ^**b**^	**0.18**
Depression		Yes, n (%)	414	13 (17.8)	52 (15.2)	.713^b^	0.03
Parkinson		Yes, n (%)	414	5 (6.8)	26 (7.6)	1.00^c^	0.01
Hypertension		Yes, n (%)	414	46 (63.0)	220 (64.5)	.914^b^	0.01

Definitions of abbreviations: *CCI* Charlson Comorbidity Index, *DSS* Dementia Screening Scale, *ES* Effect size, *IQR* Interquartile range, *MNA-SF* Mini Nutritional Assessment Short-Form, *NPI-Q* Neuropsychiatric Inventory-Questionnaire, *NRS* Numerical Rating Scale, *p* p value, *PAINAD* Pain Assessment in Advanced Dementia, *PSMS* Physical Self-Maintenance Scale, *SD* Standard deviation, *SGB XI* German Social Code XI; values in **bold **indicate statistically significant results (*p* ≤ 0.05) and the corresponding effect estimates

^a^ Calculation performed using the independent samples t test (effect size: Cohen’s d)

^b^ Calculation performed using the chi-square test (χ² test) (Effect size: Cramér’s V)

^c^ Calculation performed using Fisher’s exact test (Effect size: Cramér’s V)

^d^ Calculation performed using the Mann‒Whitney U test (effect size: r)

^e^ With the use of hearing or vision aids

^f^ Definitions include bed rails, belt restraints, and other mechanical measures

### Factors associated with the prevalence of delirium

In the multivariate regression analysis, the following variables remained significantly associated with the prevalence of delirium in nursing home residents (Table 3): duration of residence in the current nursing home for 25 months or longer, prevalent pain behavior (PAINAD), greater severity of neuropsychiatric symptoms (NPI-Q), and a documented diagnosis of dementia.

**Table 3:** Factors associated with the prevalence of delirium according to univariate and multivariate analyses

Independent variable^a^	Measurement^b^	Univariate regression		Multivariate regression^c^	
		*p*	OR (95% CI)	*p*	AOR (95% CI)
(Intercept)	**-**	**-**	**-**	0.005	**-**
Age	Years	0.731	1.00 (0.98–1.03)	0.685	1.01 (0.97–1.05)
Sex of resident	Male	0.141	0.64 (0.35–1.14)	0.485	0.75 (0.34–1.67)
Time as resident	≥ 25 months	**0.009**	**2.55 (1.32–4.93)**	**0.044**	**2.74 (1.08–6.94)**
PSMS score	Score 6–30	**< 0.001**	**1.22 (1.13–1.32)**	0.053	1.14 (1.01–1.30)
MNA-SF cutoff	Risk for malnutrition: Score 8–11	**0.002**	**3.12 (1.61–6.04)**	0.913	1.05 (0.45–2.46)
	Malnutrition: Score 0–7	**< 0.001**	**7.38 (3.51–15.55)**	0.997	1.00 (0.37–2.75)
PAINAD cutoff	Probable pain: Score ≥ 2	**< 0.001**	**5.49 (2.46–12.25)**	**0.009**	**3.67 (1.51–8.93)**
NPI-Q severity score	Score 0–36	**< 0.001**	**1.26 (1.15–1.37)**	**0.016**	**1.17 (1.04–1.32)**
Dementia	Yes	**0.016**	**2.51 (1.24–5.09)**	**0.027**	**2.01 (1.12–3.59)**
Neuroleptics	Yes	**< 0.001**	**2.70 (1.93–3.76)**	0.395	1.36 (0.68–2.71)

Definitions of abbreviations: *4AT* 4 ‘A’s” test, *AOR* Adjusted odds ratio, *AUC* Area under the curve, *CI* Confidence interval, *MNA-SF* Mini Nutritional Assessment Short-Form, *NPI-Q* Neuropsychiatric Inventory-Questionnaire, *OR* Odds ratio, *p* p value, *PAINAD* Pain Assessment in Advanced Dementia, *PSMS* Physical Self-Maintenance Scale, *VIF* Variance inflation factor; values in **bold **indicate statistically significant results (*p* ≤ 0.05) and the corresponding effect estimates

^a^ *Dependent variable*: Probable delirium as determined with the 4AT (yes: score ≥ 4; no: score ≤ 3)

^b^ *Reference categories*: Sex of the resident: female; Time as a resident: ≤ 24 months; MNA-SF cutoff: score 12–14 (normal nutrition); PAINAD cutoff: ≤ 1 (no pain indicated); Dementia: no; Neuroleptics: no

^c^ Model sample size: *n* = 351; Nagelkerke Pseudo R^2^: 0.42; VIF: 1.02–1.12; AUC: 0.87; specificity: 96.1%; sensitivity: 38.2%; Brier score: 0.10

## Discussion

This study aimed to determine the prevalence of delirium and its subtypes among nursing home residents in North Rhine-Westphalia, Germany, as well as to identify associated factors for delirium in this population. The current multicenter cross-sectional study was conducted using validated assessments administered by trained nursing staff. The prevalence of delirium was 17.6% (95% CI: 13.4–22.7) among 415 residents from 33 nursing homes. Nonmotor delirium (43.1%) was the most common subtype, whereas the mixed subtype (13.9%) was the least common. Time as a resident in the current nursing home (≥ 25 months), pain behavior assessed by the PAINAD, severity of neuropsychiatric symptoms measured by the NPI-Q, and a diagnosis of dementia were associated with the prevalence of delirium in residents.

### Prevalence of delirium

The prevalence of delirium in older nursing home residents, according to a systematic review, ranges widely from 1.0 to 57.9%. This variation was attributed to differences in delirium detection methods and the prevalence of dementia across studies [18]. According to studies from the past decade with comparable designs, the prevalence of delirium reported in the present study (17.6%) aligns closely with that reported in a Belgian study (14.2%) [19], in which trained research nurses utilized the Delirium Observation Screening Scale (DOSS) for detection. In contrast, notably higher prevalence rates have been reported in two Italian studies that also applied the 4AT, which was administered by physicians and nurses, yielding rates of 36.8% [17] and 27.2% [27].

Although the 4AT was originally designed to be used without special training [24], a recent survey of healthcare practitioners has suggested that even basic instruction could increase screening effectiveness [41]. Therefore, all the raters in the present study received training on delirium and the application of the 4AT, including the differentiation from dementia. Another factor to consider is the variation in dementia rates. Dementia is a well-established risk factor for delirium [16, 42, 43], and higher prevalence rates of delirium have consistently been reported among residents with dementia. For example, McCusker et al. [16] reported a delirium prevalence of 16.5% among dementia residents, compared with only 2.1% in those without dementia. The Italian studies reported dementia rates of 51.9% [17] and 44.0% [27], whereas the Belgian study has reported a dementia rate of 27.5% [19]. These findings may partly explain why the prevalence of delirium in the present study, which had a dementia rate of 40.3%, was lower than that reported in the Italian studies [17, 27] but higher than that reported in the Belgian study [19]. No further relevant methodological or clinical differences were identified. Overall, the findings of the present study confirm those of previous research indicating that delirium is a widespread health issue in nursing homes.

### Prevalence of delirium subtypes

There is also a lack of studies that investigated delirium motor subtypes in nursing homes. To the best of our knowledge, only two Italian studies [17, 27] have examined the prevalence of these subtypes using the DMSS. One of the study reported that the nonmotor (26.9%) and mixed (26.9%) subtypes were the most common [17], whereas the other study reported that the hyperactive subtype (35.4%) was the most common, followed by the nonmotor subtype (30.4%) [27]. In contrast, the present study revealed that the nonmotor subtype was the most common subtype (43.1%), followed by the hypoactive subtype (25.0%).

These results suggest that the nonmotor subtype may play a more significant role in nursing homes than in other settings, particularly acute care, where it has the lowest prevalence among all delirium subtypes [44]. One possible explanation for this finding could be that individuals with the nonmotor subtype differ in terms of clinical vulnerability compared with those with other delirium subtypes. Accordingly, limited evidence suggests that individuals with the nonmotor subtype show better functional performance and experience an overall milder course of delirium than those with other delirium subtypes [44–47]. However, more knowledge is needed to understand the factors influencing these setting-related differences.

### Associated factors

As previously outlined, dementia is considered one of the most significant predisposing factors for the development of delirium [14]. Accordingly, dementia is also recognized as a key factor associated with the prevalence of delirium in nursing homes [16, 17, 42, 43, 48]. The findings of the present study confirmed this association. In this context, it is notable that greater severity of neuropsychiatric symptoms, as measured by the NPI-Q, was significantly associated with the prevalence of delirium. The symptoms assessed by the NPI-Q, such as agitation/aggression and nighttime disturbances [36], are typical behavioral symptoms in dementia but may also indicate delirium [49]. Given that dementia remained associated with delirium in the regression model, the increased severity of NPI-Q symptoms cannot be fully explained by dementia alone.

Neuroleptics, which are commonly used to treat behavioral symptoms associated with dementia and as symptomatic therapy for delirium [50], were not associated with the prevalence of delirium according to the multivariate analysis conducted herein. While some studies in the nursing home setting have identified such an association [17, 27, 48], others have not [19, 51]. A possible explanation for this could lie in the distribution and characteristics of delirium subtypes. In the present study sample, the nonmotor and hypoactive subtypes predominated. Studies conducted across various settings have suggested that these two subtypes may present with less pronounced symptoms, including milder delusions, motor agitation, and affective lability [46, 47, 52, 53], may be less frequently identified by nurses [54], and may be less likely to receive psychopharmacological treatment [55–58]. Given that the hyperactive and mixed subtypes occurred less frequently in the present study sample, this might explain why the use of neuroleptics was not significantly associated with the prevalence of delirium according to the multivariate analysis. Although no causal conclusions can be drawn from this study, the findings support a therapeutic role of neuroleptics rather than a delirium-inducing effect.

Pain-related behavior, as measured by the PAINAD, was identified as a factor associated with delirium, which is consistent with findings from another study in the nursing home setting [48]. Evidence primarily from clinical populations suggests that pain itself is more strongly associated with the prevalence of delirium than the administration of analgesics [48, 59, 60]. Moreover, a previous study has indicated that insufficient pain medication may contribute to the onset of delirium [61]. These findings support the role of pain as an independent trigger for delirium [14] and underscore the importance of comprehensive pain management within delirium care.

Finally, the present study revealed an association between longer residence in nursing homes (≥ 25 months) and the prevalence of delirium. This may be interpreted as an indirect indicator of increasing vulnerability among residents, as longer nursing home residence is typically associated with older age, progressing functional decline, and increased morbidity – all of which are known risk factors for delirium [14]. In summary, the present results confirmed the multifactorial nature of delirium and suggested that dementia and pain-related behavior may play important roles in its development.

### Strengths and limitations

To the best of our knowledge, this is the first study in Germany to estimate the prevalence of delirium, its motor subtypes, and associated factors among nursing home residents. Cross-sectional data were analyzed from 415 randomly selected residents in 33 nursing homes across the federal state North Rhine-Westphalia. Delirium identification was performed using the 4AT, which has good to very good diagnostic properties [25, 62], alongside additional standardized tools for assessing associated factors. The data were collected by trained nursing staff, and a published study protocol ensured transparency [22].

Nevertheless, several limitations should be considered. First, statistical and analytical limitations warrant consideration. Although the initially targeted sample size of 650 residents was not fully reached, the final sample of 415 was sufficient for reliable delirium prevalence estimation. Despite the inclusion of fewer variables than originally anticipated in the final multivariate model, the reduced sample size may have resulted in wider confidence intervals and limited statistical power, particularly for detecting small to moderate associations [23]. Furthermore, group comparisons were not adjusted for multiple testing (e.g., Bonferroni correction) due to the study’s exploratory nature, which may have increased Type I error risk and potentially influenced variable selection for regression. Additionally, omitting the DSS score from regression analyses precluded exploring the relationship between cognitive impairment severity and delirium prevalence.

Second, several assessment-related limitations warrant consideration. The 4AT has shown specificity issues in populations with high dementia rates [24, 63], potentially overdiagnosing delirium, though no single screening tool is currently optimal for this group [64]. Conversely, the DMSS has lower sensitivity for nonmotor than motor subtypes [28, 65], potentially underestimating these cases. Furthermore, because the NPI-Q was developed and validated primarily in dementia populations, associations involving its severity score should be interpreted cautiously, as its psychometric properties in non-dementia populations remain insufficiently established [66]. Finally, four raters received online rather than in-person training, although all raters received the same training manual and study instructions.

Third, study design limitations warrant consideration. The single-time screening and lack of standardized assessment timing may have missed delirium cases due to its fluctuating nature. Although the use of in-house nurses may have facilitated delirium detection through their familiarity with residents’ baseline functioning, the potential for familiarity bias cannot be entirely excluded. The cross-sectional design also precludes establishing causal relationships.

Finally, selection bias may have limited sample representativeness at both the resident and institutional levels. The recruitment list initially included 30 randomly selected residents per nursing home, all of whom were visible to the raters during the recruitment process. Although residents were randomly selected, the recruitment process may have influenced the inclusion of residents for whom obtaining informed consent was easier. Furthermore, because reasons for withdrawal were not provided by all nursing homes that withdrew from the study, institutional-level selection bias cannot be excluded. Importantly, all five privately operated nursing homes initially recruited for the study withdrew, resulting in a final sample consisting exclusively of nonprofit nursing homes. As privately operated nursing homes account for 42% of German nursing homes [67], the representativeness of the final sample with regard to ownership structure may be limited.

### Implications

The high prevalence of delirium among nursing home residents underscores the need for greater awareness among all healthcare professionals, particularly nurses, who spend the most time with residents, but also with general physicians and relatives. For nurses, recognizing delirium is especially challenging when dementia is present or when hypoactive and non-motor subtypes are involved [5, 54, 68]. As nurses play a crucial role in the early detection and prevention of delirium in nursing homes, as well as in ensuring timely medical intervention, they should receive targeted delirium education during their initial training. As on-the-job training alternatives, e-learning tools offer practical solutions, as they can be seamlessly integrated into daily routine care and efficiently reach many caregivers. However, such training has rarely been evaluated in nursing homes [69], especially in German-speaking regions, thus highlighting the need for its focused development. The findings of this study may provide a useful foundation for such training.

Future research should further explore the clinical characteristics and associated factors of delirium subtypes in nursing homes, particularly the nonmotor subtype, and reasons for its potentially greater prevalence compared with acute care settings [44]. Future studies should also examine whether NPI-Q symptom profiles and their severity may help distinguish delirium from dementia in nursing homes. Moreover, simple and quick screening tools for nurses should be integrated into clinical practice. If these tools are sufficiently sensitive to rule out delirium in residents with dementia, some degree of overdiagnosis should be accepted, as the consequences of undetected delirium are considered more severe. As previously mentioned, screening methods should be refined to more accurately assess delirium in individuals with dementia [64]. Overall, delirium in the nursing home setting remains an underresearched topic. In addition to the aspects mentioned above, further studies are generally needed to investigate and confirm risk factors, outcomes, and the effectiveness of prevention and treatment strategies for nursing home residents.

## Conclusion

Nearly one in six nursing home residents (17.6%) had delirium in the present study, and almost two-thirds of these residents presented with either a nonmotor or hypoactive subtype. Dementia and pain-related behavior were identified as important associated factors. The present findings underscore the crucial role of nursing home staff in recognizing delirium. Given the serious consequences of untreated delirium, the implementation of low-threshold training programs focused on early identification and prevention is essential. Therefore, further research is needed to develop and evaluate training specifically tailored to the nursing home setting. Additionally, research should focus on enhancing the understanding of delirium subtypes, particularly the nonmotor subtype. Finally, the routine use of simple, rapid delirium screening by nurses should be integrated into routine care.

## Supplementary Information

Supplementary Material 1

## Data Availability

The dataset generated during this study is available upon reasonable request. Please contact the data management of the DZNE site Witten (data-management-witten@dzne.de).

## Abbreviations

4AT: 4 ‘A’s Test
ADLs: Activities of daily living
AOR: Adjusted odds ratio
CI: Confidence interval
DMSS: Delirium Motor Subtype Scale
DSS: Dementia Screening Scale
ICD-10-GM: International Classification of Diseases, 10th Revision, German Modification
IQR: Interquartile range
MNA-SF: Mini Nutritional Assessment Short-Form
NPI-Q: Neuropsychiatric Inventory-Questionnaire
PAINAID: Pain Assessment in Advanced Dementia
PSMS: Physical Self-Maintenance Scale
SD: Standard deviation
χ² test: Chi-square test
