# Supplementary material for: Prevalence of delirium in German nursing homes: A cross-sectional study
Source: BMC Geriatr. 2026 Jul 10;26:938. doi: 10.1186/s12877-026-07925-6 (PMC13355355; doi:10.1186/s12877-026-07925-6)
Supplement: Supplementary file 1 — Supplementary Material 1 [file 12877_2026_7925_MOESM1_ESM.docx]

**Supplementary material A: Sensitivity analysis of delirium prevalence rates across two data collection periods***

|  |  | ***5-day sample (n = 394)*** | | ***2-week sample (n = 415)*** | |
| --- | --- | --- | --- | --- | --- |
| **Variables** | **Measurement** | **n** | **Proportion (95% CI)** | **n** | **Proportion (95% CI)** |
| 4AT cutoff | Probable delirium: ≥ 4 | 67 | 17.0 (13.5-20.9) | 73 | 17.6 (13.4-22.7) |
| DMSS nonmotor subtype | Yes | 30 | 45.5 (31.6-58.8) | 31 | 43.1 (29.8-55.9) |
| DMSS hyperactive subtype | Yes | 12 | 18.2 (11.9-25.0) | 13 | 18.1 (12.0-24.4) |
| DMSS hypoactive subtype | Yes | 17 | 25.8 (15.8-35.0) | 18 | 25.0 (15.6-33.3) |
| DMSS mixed subtype | Yes | 7 | 10.6 (4.3-17.5) | 10 | 13.9 (6.5-21.3) |

***** The table above presents the two samples based on the 5-day (n = 394) and 2-week (n = 415) data collection periods. The overlapping confidence intervals for the delirium proportions indicate that there are no significant differences between the two samples with respect to any of the presented variables.

*Definitions of abbreviations:* 4AT = 4 “A’s” Test; CI = confidence interval; DMSS = Delirium Motor Subtype Scale.

**Supplementary material B: Sensitivity analysis of sample characteristics across two data collection periods***

|  | |  | ***5-day sample (n = 394)*** | | ***2-week sample (n = 415)*** | |
| --- | --- | --- | --- | --- | --- | --- |
| **Variables** | | **Measurement** | ***p*** | **ES (95% CI)** | ***p*** | **ES (95% CI)** |
|  | **Sociodemographic characteristics of sample** | | | | | |
| Age | | Years | .962^a^ | -0.01 (-0.27-0.26) | .775^a^ | 0.03 (-0.22-0.29) |
| Sex of resident | | Female  Male | .120^b^ | 0.09 (<0.01-0.18) | .203^b^ | 0.07 (<0.01-0.16) |
| Family status | | Widowed | .512^b^ | 0.08 (<0.01-0.17) | .513^b^ | 0.07 (<0.01-0.17) |
|  |  | Divorced |  |  |  |  |
|  |  | Single |  |  |  |  |
|  |  | Married, relationship |  |  |  |  |
| Length of stay in current nursing home | | ≤ 24 months | <.001^b^ | 0.19 (0.09-0.29) | .001^b^ | 0.18 (0.08-0.27) |
|  |  | ≥ 25 months |  |  |  |  |
| Hospital stay(s) < 3 months | | Yes | .996^b^ | 0.01 (<0.01-0.11) | 1.00^b^ | <0.01 (<0.01-0.10) |
|  | **Health and functional assessments** | | | | | |
| DSS score | | Score 0-12 | <.001^d^ | 0.41 (0.32-0.49) | <.001^d^ | 0.43 (0.35-0.50) |
| DSS cutoff | | Mild to severe dementia: ≥ 3 | <.001^c^ | 0.27 (0.18-0.36) | <.001^c^ | 0.29 (0.20-0.37) |
| PSMS score | | Score 6-30 | <.001^a^ | 1.08 (0.80-1.35) | <.001^a^ | 1.00 (0.74-1.26) |
| MNA-SF cutoff | | Normal nutrition | <.001^b^ | 0.25 (0.15-0.34) | <.001^b^ | 0.25 (0.16-0.34) |
|  |  | Risk for malnutrition |  |  |  |  |
|  |  | Malnutrition |  |  |  |  |
| PAINAD score | | Score 0-10 | <.001^d^ | 0.31 (0.20-0.42) | <.001^d^ | 0.33 (0.22-0.42) |
| PAINAD cutoff | | Probable pain: ≥ 2 | <.001^b^ | 0.31 (0.21-0.40) | <.001^b^ | 0.33 (0.24-0.41) |
| NRS (Pain) score | | Score 0-10 | .759^d^ | 0.02 (<0.01-0.12) | .779^d^ | 0.01 (<0.01-0.12) |
| NPI-Q symptom score | | Score 0-12 | <.001^d^ | 0.27 (0.17-0.37) | <.001^d^ | 0.31 (0.21-0.40) |
| NPI-Q severity score | | Score 0-36 | <.001^d^ | 0.30 (0.20-0.41) | <.001^d^ | 0.34 (0.24-0.43) |
|  | **Clinical and care-related characteristics** | | | | | |
| Vision impairment^e^ | | Yes | .837^b^ | 0.02 (<0.01-0.12) | .866^b^ | 0.02 (<0.01-0.11) |
| Hearing impairment^e^ | | Yes | .327^b^ | 0.06 (<0.01-0.16) | .182^b^ | 0.07 (<0.01-0.17) |
| Fall(s) < 3 months | | Yes | .227^b^ | 0.07 (<0.01-0.17) | .206^b^ | 0.07 (<0.01-0.17) |
| Physical restraint < 4 weeks^f^ | | Yes | .595^c^ | 0.06 (<0.01-0.15) | .596^c^ | 0.06 (<0.01-0.15) |
| Feeding tube | | Yes | .594^c^ | 0.05 (<0.01-0.15) | .592^c^ | 0.05 (<0.01-0.15) |
| Urinary catheter | | Yes | .326^c^ | 0.06 (<0.01-0.15) | .327^c^ | 0.06 (<0.01-0.15) |
| CCI score | | Score 0-24 | .197^d^ | 0.07 (<0.01-0.16) | .310^d^ | 0.05 (<0.01-0.14) |
| Neuroleptics | | Yes | <.001^b^ | 0.19 (0.10-0.29) | <.001^b^ | 0.19 (0.09-0.28) |
| Dementia | | Yes | .002^b^ | 0.17 (0.07-0.26) | .001^b^ | 0.18 (0.08-0.27) |
| Depression | | Yes | .980^b^ | 0.01 (<0.01-0.11) | .713^b^ | 0.03 (<0.01-0.12) |
| Parkinson | | Yes | 1.00^c^ | <.01 (<0.01-0.10) | 1.00^c^ | 0.01 (<0.01-0.11) |
| Hypertension | | Yes | 718^b^ | 0.03 (<0.01-0.12) | .914^b^ | 0.01 (<0.01-0.11) |

***** The table above presents the two samples based on the 5-day (n = 394) and 2-week (n = 415) data collection periods. The p values refer to group comparisons between residents with and without delirium within each respective sample. The overlapping confidence intervals for the effect sizes indicate that there are no significant differences between the two samples with respect to any of the presented variables.

*Definitions of abbreviations:* CCI = Charlson Comorbidity Index; CI = confidence interval; DSS = dementia screening scale; ES = effect size; MNA-SF = Mini Nutritional Assessment Short-Form; NPI-Q = Neuropsychiatric Inventory-Questionnaire; NRS = numerical rating scale; p = p value; PAINAD = Pain Assessment in Advanced Dementia; PSMS = Physical Self-Maintenance Scale; SGB XI = German Social Code XI.

^a^ Calculation performed using the independent samples t test (effect size: Cohen’s d)

^b^ Calculation performed using the chi-square test (χ² test) (Effect size: Cramér’s V).

^c^ Calculation performed using Fisher’s exact test (Effect size: Cramér’s V).

^d^ Calculation performed using the Mann‒Whitney U test (effect size: r).

^e^ With the use of hearing or vision aids.

^f^ Definitions include bed rails, belt restraints, and other mechanical measures.
